# P-806. How close are we to achieving the trifecta of optimal treatment duration, route of therapy, and repeat blood culture testing for uncomplicated gram-negative bacteremia?

**DOI:** 10.1093/ofid/ofae631.998

**Published:** 2025-01-29

**Authors:** Andrea H Son, Morgan K Morelli, Michelle T Hecker

**Affiliations:** The MetroHealth System, Cleveland, Ohio; MetroHealth Medical Center, Cleveland, Northern Mariana Islands; The MetroHealth System, Cleveland, Ohio

## Abstract

**Background:**

A 2021 consensus document outlined recommendations for treatment duration, oral antibiotic use, and need for repeat blood cultures in patients with uncomplicated gram-negative bacteremia. Data support 7 days of effective therapy and oral step-down therapy in clinically stable patients. Repeat blood cultures were not recommended except for patients with concern for endovascular infection. We sought to evaluate the percentage of our gram-negative bacteremia cases that would be defined as uncomplicated and how well our practices for these cases aligned with the 2021 recommendations

**Table 1.**
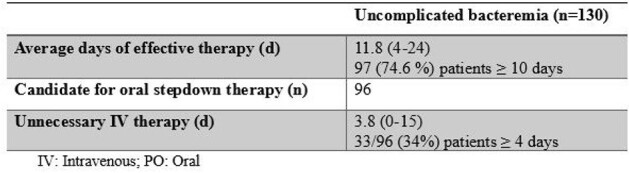
Antibiotic days of therapy

**Methods:**

This retrospective cohort study included adult patients hospitalized between 1/1/2022 and 12/31/22 who had a blood culture with growth of a gram-negative organism and who received ≥ 4 days of antibiotics for the bacteremia. Effective therapy required both use of an antibiotic to which the organism was susceptible and source control. Unnecessary IV therapy was defined as continuation of IV therapy in patients with clinical stability for ≥ 24 hours who had an oral treatment option available.

**Table 2.**
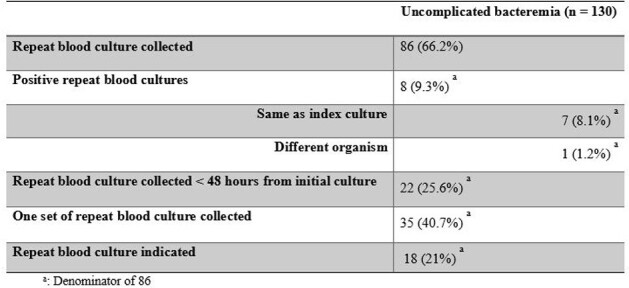
Repeat blood cultures

**Results:**

During 2022, 251 unique patients were hospitalized with gram-negative bacteremia and received ≥ 4 days of antibiotics, 130 (52%) of whom were classified as having uncomplicated bacteremia. The average duration of effective therapy was 11.8 days and 74.6% of patients received ≥ 10 days. Unnecessary IV therapy was continued for an average of 3.8 days and 33 (34%) were not transitioned to an orally available option by day 4 of clinical stability. Repeat blood cultures were done in 86 (66%) patients but were indicated in only 18 (14%). Twenty-two (26%) repeat blood cultures were collected < 48 hours after the initial positive blood culture

**Conclusion:**

Using the 2021 consensus recommendations as a guide, more than half of patients with uncomplicated gram-negative bacteremia at our institution received longer than recommended durations of therapy, were not transitioned to an oral option when clinically stable and had repeat blood cultures done when not indicated. A multifaceted intervention to achieve the trifecta of optimal treatment duration, route of therapy, and repeat blood culture testing for uncomplicated gram-negative bacteremia is planned.

**Disclosures:**

**All Authors**: No reported disclosures

